# Can Tongue Acupuncture Enhance Body Acupuncture? First Results from Heart Rate Variability and Clinical Scores in Patients with Depression

**DOI:** 10.1155/2014/329746

**Published:** 2014-03-23

**Authors:** Xian Shi, Huan Wang, Lu Wang, Zengkai Zhao, Daniela Litscher, Jingqiao Tao, Ingrid Gaischek, Zemin Sheng, Gerhard Litscher

**Affiliations:** ^1^Department of Acupuncture, People's Liberation Army General Hospital, Beijing 100853, China; ^2^Research Unit for Complementary and Integrative Laser Medicine, Research Unit of Biomedical Engineering in Anesthesia and Intensive Care Medicine, and TCM Research Center Graz, Medical University of Graz, Auenbruggerplatz 29, 8036 Graz, Austria; ^3^Private Clinic Laßnitzhöhe, 8301 Laßnitzhöhe, Austria

## Abstract

Tongue acupuncture (TA) is a method which is not used in western medicine and even in China it is applied very rarely in clinical practice. This study aimed at investigating whether additional TA can improve the efficacy of body acupuncture (BA) in patients with depression. Twenty patients with a mean age of ± SD of *42.9* ± 11.2 years were randomly divided into two groups (*n* = 10 patients each), one group receiving BA (Zusanli, Sanyinjiao, Neiguan, Shenting, Yintang, and Baihui) and the other receiving BA and TA (Liver, Heart, and Brain). The quantitative and qualitative outcome measures were heart rate (HR), heart rate variability (HRV), and different clinical scores. We found that in both groups all scores and HR improved significantly, whereas HRV increased partly significantly. It seems that TA can enhance acute and treatment effects of BA in patients with depression. The investigation of de qi sensation in TA needs further attention.

## 1. Introduction

Tongue acupuncture (TA) is a unique natural therapy. Specific tongue acupoints are supposed to be related to various functional domains of the body. It is claimed that TA can stimulate different meridians associated with different organs' functions in order to adjust blood circulation and energy-flow in the body [1].

De qi sensation evoked by TA is essential to its clinical effectiveness. One purpose of TA is restoring consciousness and brain resuscitation. In China, it is used as a complementary method to treat not only several diseases, like stroke [2], but also children with visual impairment [3].

In contrast to TA, there are a lot of clinical investigations concerning body acupuncture (BA) and major depressive disorders [4].

**Figure 1:**
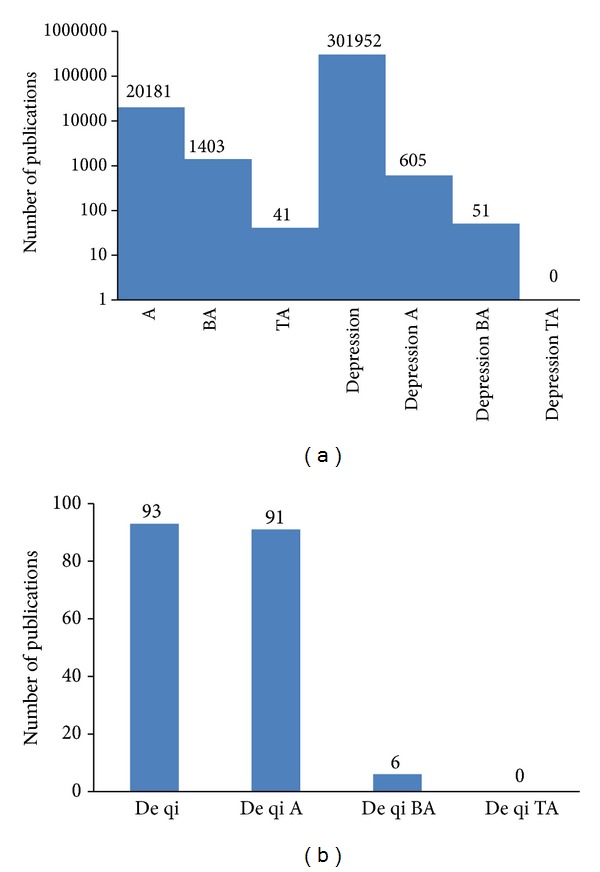
Use of different acupuncture approaches (http://www.pubmed.gov/): A … acupuncture; BA … body acupuncture; and TA … tongue acupuncture.

Figures 1(a) and 1(b) summarize the number of publications concerning different terms related to TA and BA.

Our research group recently found acute stimulation effects on neurovegetative parameters like heart rate (HR) and heart rate variability (HRV) in patients with depression [5] and insomnia [6].

The present clinical study was performed at the Military Acupuncture Centre at the People's Liberation Army General Hospital, Beijing, in cooperation with the Traditional Chinese Medicine (TCM) Research Center Graz, Austria (http://litscher.info/ and http://tcm-graz.at/). This paper compares results from BA and BA + TA measurements in depression patients with regard to neurophysiological parameters like HR and HRV.

## 2. Materials and Methods

### 2.1. Patients

Twenty patients (all females; mean age ± SD 42.9 ± 11.2 years; range 20–65 years) suffering from depression (Chinese diagnosis “Yu Zheng”) received acupuncture treatment at the Chinese People's Liberation Army Hospital, Beijing. The clinical evaluation of the patients, performed before the first and after the last acupuncture session, used three main scales: the Hamilton Anxiety Rating Scale (HAM-A) [7], the Athens Insomnia Scale (AIS) [8], and the Hamilton Rating Scale for Depression (HRSD) [9]. No patient was under the influence of centrally active medication or had a history of heart or cerebrovascular disease, respiratory or neurological problems, or hypertension. The study was approved by the Ethics Committee of the Chinese People's Liberation Army Hospital and carried out in compliance with the Declaration of Helsinki. All patients gave oral informed consent.

### 2.2. Teleacupuncture

Electrocardiographic (ECG) biosignal recording was performed in China, and data analysis took place in Europe. For ECG registration three adhesive electrodes (Skintact Premier F-55, Leonhard Lang GmbH, Innsbruck, Austria) were used which were applied to the chest.

The research team in China used a medilog AR12 HRV (Huntleigh Healthcare, Cardiff, United Kingdom) system from the TCM Research Center at the Medical University in Graz. The system has a sampling rate of 4096 Hz; the raw data are stored digitally and transferred to the TCM Research Center Graz via the Internet. The biosignals were analyzed and HRV was calculated.

Like in previous studies [5, 6, 10], mean HR, total HRV, and the LF (low frequency)/HF (high frequency) ratio of HRV were chosen as evaluation parameters, as such being recommended by the Task Force of the European Society of Cardiology and the North American Society of Pacing and Electrophysiology [11].

### 2.3. Body Acupuncture, Tongue Acupuncture, and Procedure

**Figure 2:**
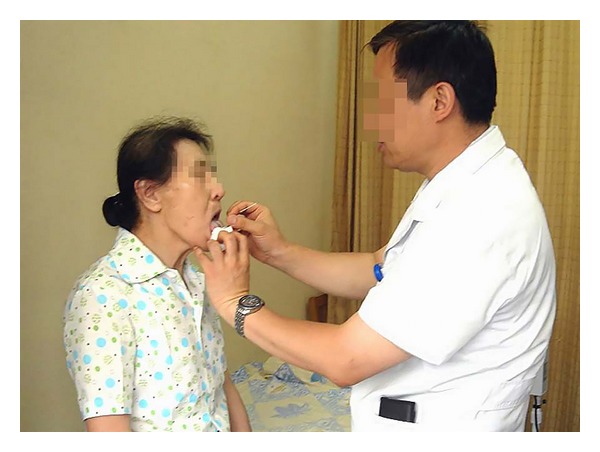
Tongue acupuncture at the Military Acupuncture Training Centre at the People's Liberation Army General Hospital, Beijing.

The patients were randomly divided into two groups using a simple randomization (numbers by chance). One group (*n* = 10; mean age 39.2 ± 13.2 years; range 20–65 years) received BA in six sessions and the other group (*n* = 10; mean age 46.6 ± 7.8 years; range 38–60 years) additionally received TA (see Figure 2) in all six sessions. HR-HRV recordings were performed during the first and the last acupuncture session.

The following body acupoints were used in this study: Zusanli (ST36,bilateral), Sanyinjiao (SP6, bilateral), Neiguan (PC6, bilateral), Shenting (GV24), Yintang (Ex-HN3), and Baihui (GV20). The sterile, single-use needles (0.30 × 25 mm, Huan Qiu, Suzhou, China) were inserted perpendicularly, and the needle was stimulated clockwise and counterclockwise for 15 seconds each, with six rotations per second, resulting in 90 rotations per stimulation.

**Figure 3:**
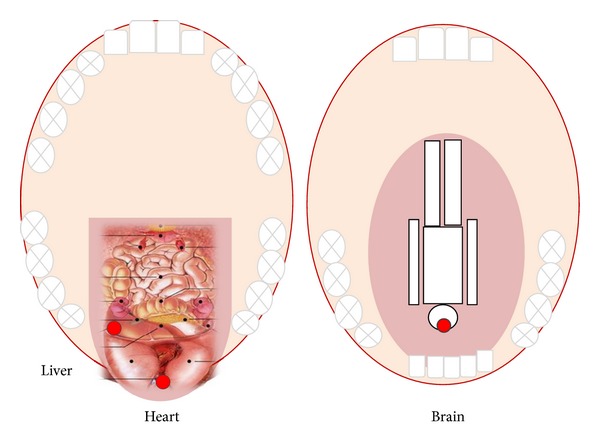
Tongue acupuncture points used in this study.

The patients in the TA group received TA in every acupuncture session, immediately before BA. For TA, the points Liver, Heart, and Brain (Figure 3) were always used in this order [1]. The needle was inserted obliquely into the tongue acupuncture point, to a depth of 0.5–1 cun, and immediately removed again; then the next tongue point was treated likewise.

**Figure 4:**
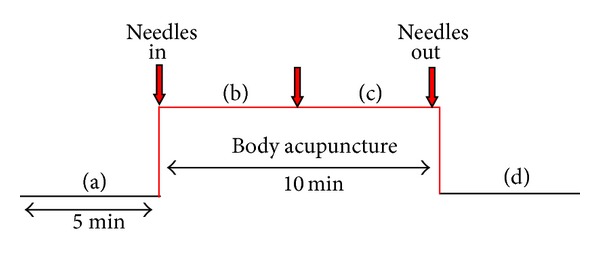
Measurement procedure for body acupuncture.

The measurement profile and measurement phases (a–d) of the BA treatment are shown in Figure 4. Four registration periods (5 min each) were compared: one before acupuncture (a), two during acupuncture (b, c), and one as a control after removing the needles (d). In addition, blood pressure was measured at the beginning and at the end of the acupuncture sessions.

### 2.4. Statistical Analysis

Data were analyzed using SigmaPlot 12.0 software (Systat Software Inc., Chicago, USA). Graphical presentation of results uses box plot illustrations. Testing was performed with Friedman repeated measures ANOVA on ranks and Tukey or Holm-Sidak test. The criterion for significance was *P* < 0.05.

## 3. Results

**Figure 5:**
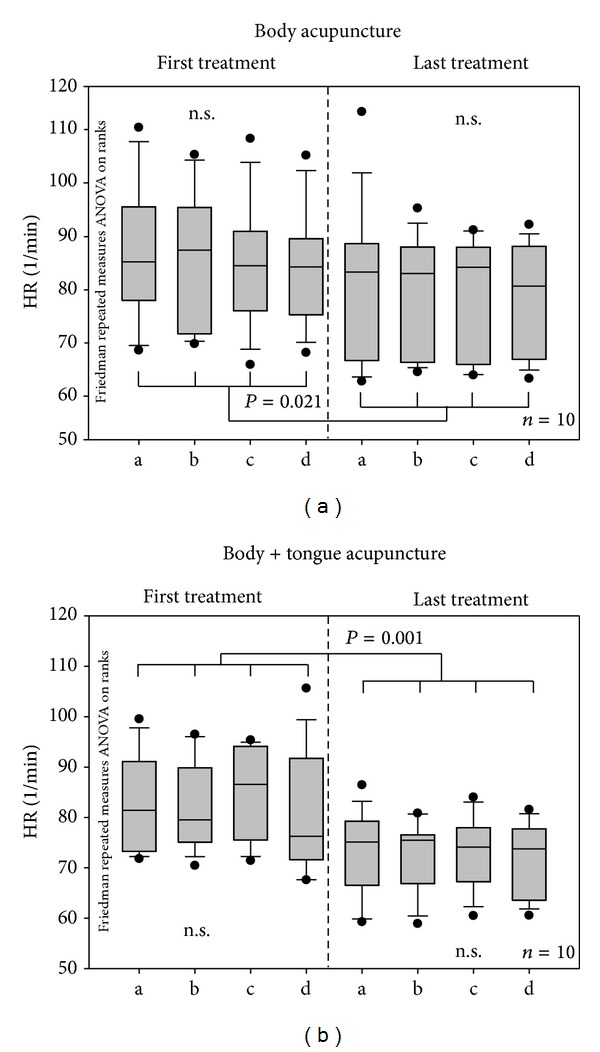
Box plots displaying the changes in mean heart rate (HR). Within the single treatment sessions, no significant effects could be found. When comparing the values of the first session to those of the last session, however, HR had decreased significantly. The ends of the boxes define the 25th and 75th percentiles with a line at the median and error bars defining the 10th and 90th percentiles.

**Figure 6:**
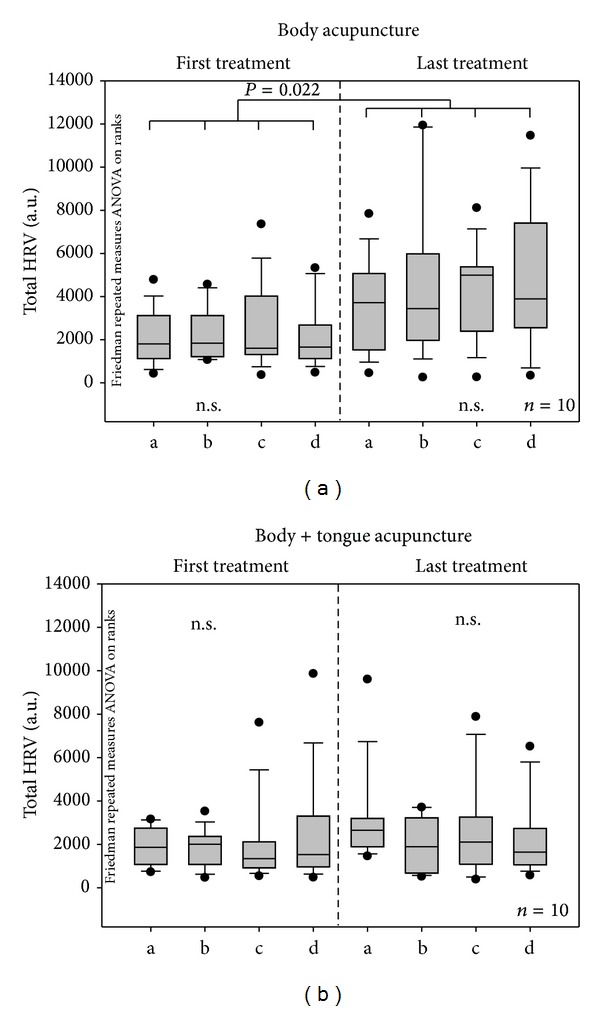
Changes in total heart rate variability (HRV). For further explanations, see Figure 5.

Figures 5 and 6 show the mean HR and total HRV from the ECG recordings of both patient groups during the first and last treatment session, respectively. HR had decreased significantly in both groups in the course of the treatment.

In contrast to HR, HRV had increased significantly in the BA group during the course of the treatment (see Figure 6).

**Figure 7:**
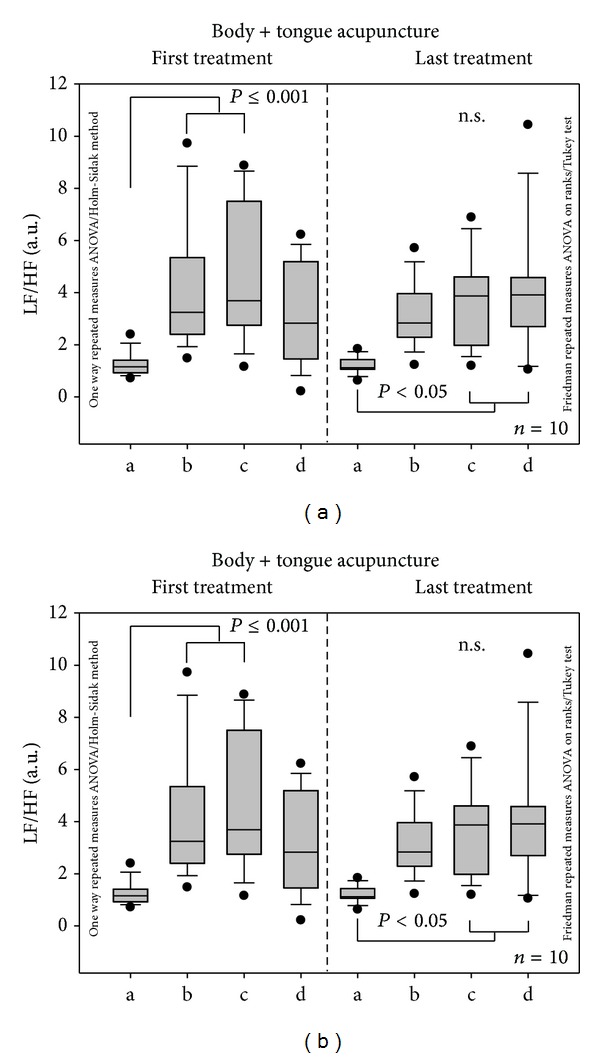
The low frequency (LF)/high frequency (HF) ratio did not change significantly when comparing the first and last treatment. For further explanations, see Figure 5.

Furthermore, continuous HRV monitoring showed significant alterations in the LF/HF ratio within the single treatment sessions (see Figure 7). The comparison between the first and the last treatment, however, did not reach the level of significance.

The direct statistical comparison between the TA and BA group did not yield significant changes; however, there was a marked decrease of HR in the TA group and the decrease of the HAM-A score (see Table 1) showed a higher significance (*P* = 0.008 in the BA group and *P* < 0.001 in the TA group).

The analysis of the three clinical scores revealed interesting results. In all scores there was a significant reduction (see Table 1).

The data of the blood pressure values showed insignificant results (see Table 1). The de qi sensation during TA was similar to that during BA. After the arrival of qi, the TA patients reported sensations of distension, heaviness, and numbness; in addition, these sensations spread to the throat.

## 4. Discussion

Tongue acupuncture is an innovative but not commonly used technique in the traditional Chinese medicine [12]. It originated from the theory of TCM through scientific research. Forty points on the tongue were discovered that correspond to organs and certain parts of the body [1]. Studies showed that TA and BA can improve the visual status of children with visual disorders, both peripheral and central in origin [3]. The authors of a rat model study monitored the evoked activity of the digastric electromyography elicited by electrical stimulation of the tongue [13]. In a study dealing with aphasia after stoke, authors mentioned that tongue bleeding, deep insertion, and strong stimulation were adopted by many practitioners [14]. Li et al. found that tongue acupuncture has a better therapeutic effect on stroke [2]. There are also a few other studies available in the database PubMed which also deal with the topic tongue acupuncture; however, there are no publications available in connection with depression or de qi sensation (Section 1).

In Asia and also in Europe, depression is one of the most disabling diseases, causing a significant burden both to the individual and to society [15–17]. Detailed information on this important topic can be found in the discussion section of one of our previous publications published recently [15]. As stated in that article, one important way to stop this cost explosion in China and Europe is through increased research efforts in this field. Better detection, prevention, treatment, and patient management are imperative to reduce the burden of depression and its cost [15–17].

As previously described, continuous electrical auricular acupuncture is one special kind and new method to treat patients with neurological diseases like depression [15]. The results of our present study are in accordance with previous investigations [15]. All clinical scores (HAM-A, AIS, and HRSD) showed a significant improvement already after 6 TA/BA and BA sessions. However, it has to be mentioned that the baseline values of the HAM-A score differed between the two treatment groups because randomization was performed by chance and not by score assessment. In addition, HR and HRV, which are reliable indicators of the state of health [5, 6, 15], also improved, partly significantly.

When performing TA, most acupuncturists do not leave the needle in place; they puncture the surface of the tongue and describe the fact that they receive good therapeutic effects in some clinical applications like in apoplectic aphasia rehabilitation [14]. Horizontal and deep puncturing approaches were also sometimes used. This method refers to piercing the tongue from one side to the other [18, 19]. Deep puncturing needs long needles for treatment towards the root of the tongue [20].

In conclusion, our study shows clinical (scores) and neurovegetative (HRV) improvements in parameters after BA and TA acupoint stimulation in patients with depression. It can be stated that invasive tongue stimulation with needles does not have inhibitory effects on BA; on the contrary, it seems that it can enhance acute and treatment effects of BA. The investigation of de qi sensation [21] needs further attention. Up to now, it has not been described in detail in English or even in Chinese scientific literature.

## Figures and Tables

**Table 1:** Changes in clinical scores and blood pressure values between the first (M1) and last (M2) treatment session. Values are given as mean ± SD (standard deviation).

	Body acupuncture			Body + tongue acupuncture		
	M1	M2	*P*	M1	M2	*P*
HAM-A	18.9 ± 3.9	16.6 ± 3.5	0.008	24.4 ± 7.9	19.0 ± 7.1	<0.001
AIS	15.0 ± 6.7	11.3 ± 5.1	<0.001	13.9 ± 4.0	8.3 ± 3.2	0.002
HRSD	22.2 ± 5.4	19.5 ± 4.8	0.001	22.3 ± 5.9	17.4 ± 4.8	0.002
BPsys [mmHg]	105.3 ± 9.8	103.6 ± 9.8	n.s.	113.3 ± 10.4	113.1 ± 7.4	n.s.
BPdia [mmHg]	66.4 ± 4.3	66.3 ± 4.7	n.s.	69.2 ± 6.4	69.5 ± 6.0	n.s.

HAM-A: Hamilton Anxiety Rating Scale [7]; AIS: Athens Insomnia Scale [8]; HRSD: Hamilton Rating Scale for Depression [9]; BPsys: systolic blood pressure; BPdia: diastolic blood pressure; and n.s.: not significant.
